# Environmental and inflammatory factors influencing concurrent gut and lung inflammation

**DOI:** 10.1007/s00011-024-01953-x

**Published:** 2024-10-21

**Authors:** April L. Raftery, Céline Pattaroni, Nicola L. Harris, Evelyn Tsantikos, Margaret L. Hibbs

**Affiliations:** Department of Immunology, School of Translational Medicine, Monash University, 89 Commercial Road, Melbourne, VIC 3004 Australia

**Keywords:** *Bifidobacteria*, Granulocyte colony-stimulating factor, Granulocyte signature, Gut-lung axis, Inflammatory bowel disease, Obstructive lung disease

## Abstract

**Background:**

Crohn’s disease and chronic obstructive pulmonary disease (COPD) are chronic inflammatory diseases that affect the gut and lung respectively and can occur comorbidly.

**Methods:**

Using the SHIP-1^−/−^ model of Crohn’s-like ileitis and chronic lung inflammation, the two diseases were co-investigated.

**Results:**

Contrary to prior literature, Crohn’s-like ileitis was not fully penetrant in SHIP-1^−/−^ mice, and housing in a specific pathogen-free facility was completely protective. Indeed, ileal tissue from SHIP-1^−/−^ mice without overt ileitis was similar to control ilea. However, SHIP-1^−/−^ mice with ileitis exhibited increased granulocytes in ileal tissue together with T cell lymphopenia and they lacked low abundance *Bifidobacteria*, suggesting this bacterium protects against ileitis. Lung disease, as defined by inflammation in lung washes, emphysema, and lung consolidation, was present in SHIP-1^−/−^ mice regardless of ileitis phenotype; however, there was a shift in the nature of lung inflammation in animals with ileitis, with increased G-CSF and neutrophils, in addition to type 2 cytokines and eosinophils. Deficiency of G-CSF, which protects against lung disease, protected against the development of ileitis in SHIP-1^−/−^ mice.

**Conclusions:**

These studies have defined environmental, immune, and inflammatory factors that predispose to ileitis, and have identified that comorbid lung disease correlates with a granulocyte signature.

**Supplementary Information:**

The online version contains supplementary material available at 10.1007/s00011-024-01953-x.

## Introduction

Inflammatory bowel diseases (IBD) are chronic intestinal disorders that are increasing in prevalence, with Crohn’s disease (CD) and ulcerative colitis being the two major subtypes [1]. CD is characterised by transmural and non-caseating granulomatous inflammation that can occur at any point along the gastrointestinal tract, but most commonly at the distal end of the small intestine [2]. The majority of mouse models utilised to study CD are induced colitis models [3], and thus, they do not adequately represent the inflammation in the majority of patients as these models do not develop ileitis. Furthermore, the aetiology of CD is not fully elucidated; however, it is known to be a complex interplay of genetic susceptibility, environmental factors, dysfunction of the intestinal barrier, dysbiosis of commensal microbiota, and chronic Th1/Th17 biased inflammation [4]. This highlights the need for alternative models that better represent the clinical manifestations of disease so that mechanisms can be better understood, and more effective therapies can be designed.

CD is characterised by segmented inflammation of the gastrointestinal tract with a skewing towards Th1 and Th17 responses and the presence of macrophages, neutrophils, and type 3 innate lymphoid cells that dominate the inflamed intestinal tissue [5, 6]. IL-17 and IL-23 from T cells, γδ T cells, and innate lymphoid cells can promote expression of granulocyte colony-stimulating factor (G-CSF), a key regulator of neutrophils. Neutrophils are a first line of defence against microbes that have breached the intestinal epithelial barrier but appear to lose functionality in CD patients [7]. They contribute to the formation of crypt abscesses through activation and transepithelial migration, disrupting the normal crypt architecture [8, 9], and their numbers and activation state correlate with disease severity [10]. Together, this suggests that neutrophils may represent a potential therapeutic target in CD. Eosinophils, another type of granulocyte abundantly present in the intestinal mucosa [11], are also implicated in IBD [12]. While they are thought to play a greater role in ulcerative colitis pathogenesis [12], CD patients show increased numbers of eosinophils and their chemokines [13–16] where they have been linked with active disease, flares, and stricture formation, and thus, they should not be ignored in CD studies.

Patients with CD often present with other chronic conditions, one of which is chronic obstructive pulmonary disease (COPD) [4], a progressive and incurable inflammatory lung disease characterised by airflow obstruction, chronic bronchitis, and emphysema [17]. Lung inflammation in COPD is characterised by increased numbers of alveolar macrophages, CD8 + T lymphocytes and neutrophils [17], although eosinophilic airway inflammation is also a feature of a subset of COPD patients [18]. Population studies have identified an increased prevalence of IBD in COPD patients, with these patients at increased risk of mortality [19–21]. This comorbid presentation of disease may, in part, be explained by cigarette smoking, a known risk factor for both diseases; however, the risk association between CD and COPD is higher than the risk attributed to smoking [19], suggesting other factors. Cell types that are common to both diseases, such as granulocytes, are worth investigating as their bidirectional trafficking between the gut and lung could be involved in inflammation at both sites, although this is presently unknown.

The gut-lung axis is a reciprocal interaction between the two mucosal organs with a large microbial contribution [4, 22]. The gut microbiota plays a considerable role in the development of IBD, with microbial dysbiosis regarded as a key feature of disease, and early-life antibiotics being a risk factor for IBD development [4, 23]. Generally, dysbiosis in IBD is characterised by an increase in proinflammatory bacteria and a decrease in protective species [4]. Furthermore, dysbiosis of the gut microbiota has been associated with COPD, and faecal transplant from COPD patients to mice can induce lung inflammation [24, 25]. This suggests that changes in the gut microbiota may be one mechanism by which patients with CD are more at risk of developing COPD and other chronic inflammatory lung diseases.

Mice lacking SH2 domain–containing inositol 5′ phosphatase-1 (SHIP-1) develop spontaneous CD-like ileitis with near 100% penetrance, and exhibit many key CD features including Th1/Th17 skewed transmural inflammation, marked neutrophil infiltration into ileal tissue, the formation of granulomas, and microbial dysbiosis [26–29]. In addition, clinical studies have shown that SHIP-1 activity and expression are reduced in ileal biopsies and circulating immune cells from CD patients [30–33]. Furthermore, many CD patients develop systemic extraintestinal complications [34], and some of these are observed in SHIP-1^−/−^ mice including osteoporosis [35], glomerulonephritis [36], and lung inflammation [36–39]. Collectively, this suggests that SHIP-1^−/−^ mice are a relevant model of CD [40].

Given that CD and COPD can occur comorbidly, we utilised SHIP-1^−/−^ mice that develop both gut and lung inflammation, investigating if there was a link between the two inflammatory diseases. We report that a granulocyte signature is a feature of the comorbidity of these diseases.

## Materials and methods

### Mice

**Fig. 1:**
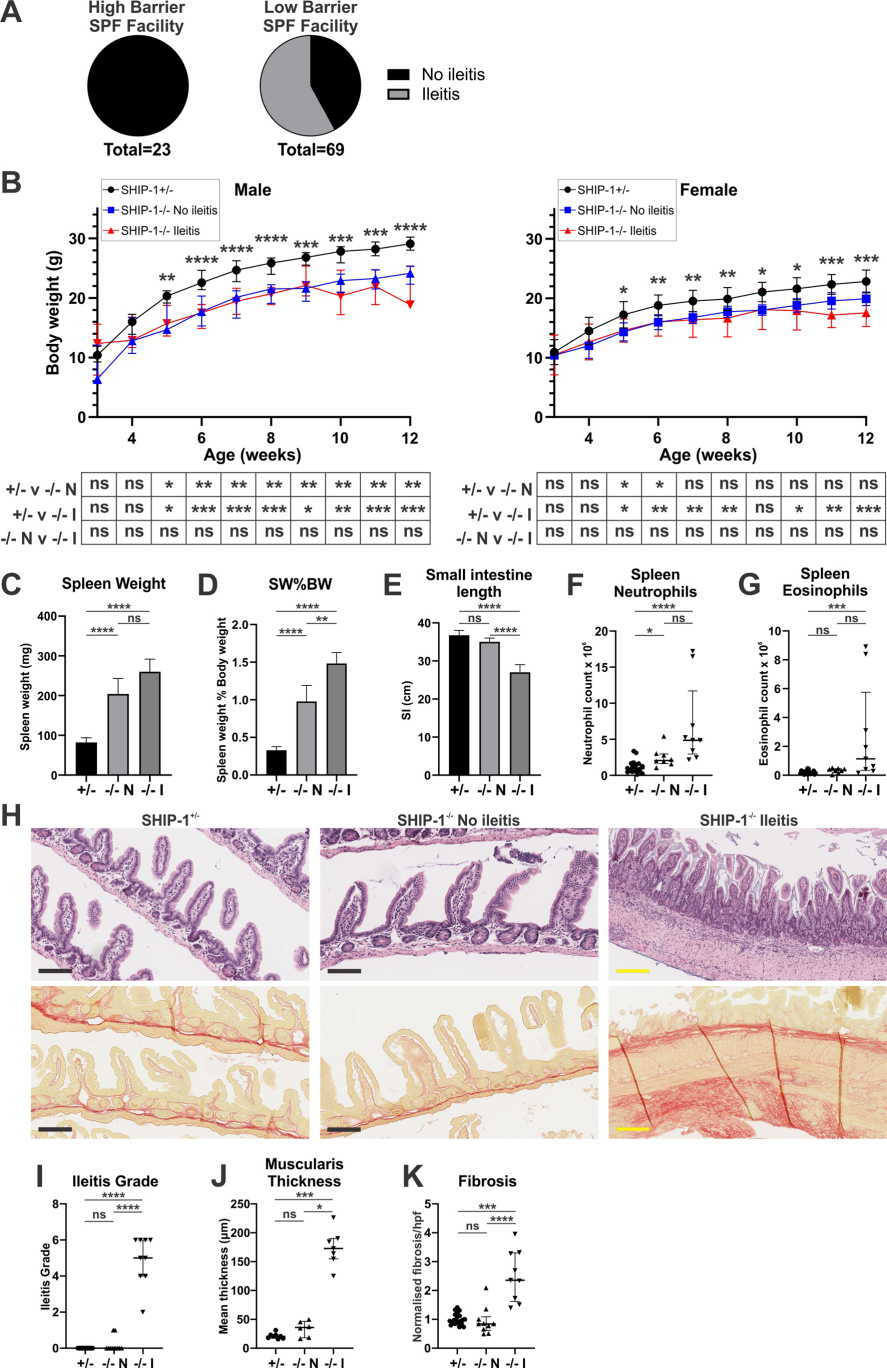
Development of ileitis in SHIP-1^-/-^ mice depends on housing environment. **A** Pie charts representing the proportion of SHIP-1^−/−^ mice that develop ileitis by 12-weeks of age in high and low barrier animal facilities. **B** Body weight (g) from 3-weeks to 12-weeks of age for littermate SHIP-1^+/−^ mice (+/-), SHIP-1^−/−^ mice without ileitis (-/- N), and SHIP-1^−/−^ mice with ileitis (-/- I). Statistics were calculated by Kruskal Wallis test (non-parametric ANOVA) with Dunn’s multiple comparisons. Results from Kruskal Wallis are presented on the graph, while those from Dunn’s multiple comparisons are outlined in tabular form under the graph. **C** Spleen weight (mg). **D** Spleen weight as a proportion of body weight. **E** Small intestine length (cm). Total number of **F** neutrophils (CD45^+^CD11b^+^Siglec-F^−^Gr-1^+^) and **G** eosinophils (CD45^+^CD11b^+^Siglec-F^+^Gr-1^−^) in spleen as determined by cell counts and flow cytometry. **H** Images of sections of Swiss-rolled ilea from representative 12-week-old mice stained with H&E (top images) or picrosirius red (bottom images). Histopathological scoring and analyses of stained ileal tissue indicating: **I** Ileitis grade; **J** Muscularis thickness (µm); and **K** Degree of fibrosis by picrosirius red staining per high power field (hpf) normalised to SHIP-1^+/−^ control mice. Data is presented as median ± IQR. ns = not significant; * *P* < 0.05; ** *P* < 0.01; *** *P* < 0.001; **** *P* < 0.0001 by Kruskal-Wallis non-parametric ANOVA test comparing SHIP-1^+/−^, SHIP-1^−/−^ without ileitis and SHIP-1^−/−^ with ileitis (**C-G**,** I-K**)

SHIP-1^−/−^ mice have been described [38] and mice on a C57BL/6 background [36] were used for these studies. Littermate SHIP-1^+/−^ mice were used as they exhibit a normal phenotype [36, 37, 41]. Double-deficient C57BL/6 background SHIP-1^−/−^G-CSF^−/−^ mice (DKO) [39] and G-CSF^−/−^ mice on a C57BL/6 background [42] were also used. Mice were bred in the specific pathogen-free (SPF) facility at the Monash Animal Research Platform, Clayton, Victoria, Australia. At weaning (3 weeks of age), they were shipped to one of two facilities at the Alfred Research Alliance, Melbourne, Victoria, Australia with differing health status according to barrier level. Studies were largely performed on young adult 10- to 12-week-old mice, except for aged analyses, which were performed on 20-week-old mice. Once housing differences in ileitis penetrance were identified (Fig. 1), only SHIP-1^−/−^ mice housed in the low barrier facility were further examined, comparing to co-housed littermate SHIP-1^+/−^ mice. This allowed the direct comparison of the immune cell compartment of SHIP-1^−/−^ mice without or with ileitis, with the mice otherwise having the same environmental influences.

Animal experiments were conducted according to National Health and Medical Research Council Australia (NHMRC) guidelines and approved by the Animal Ethics Committee of the Alfred Research Alliance, Melbourne (project numbers E1830/2018/M and E8248/2021/M).

### Bronchoalveolar lavage (BAL)

Lungs from terminally anesthetized mice were washed once with 400 µL ice-cold phosphate-buffered saline (PBS) followed by a further three washes with 300 µL ice-cold PBS. Cell counts were performed by haemocytometer and the BAL fluid (BALF) from the first lavage was stored at -80 °C until cytokines were measured by bioassay (outlined below).

### Histology

Although, the ileum is typically defined as the distal third of the small intestine, this was not accurate in the case of SHIP-1^−/−^ mice harbouring ileitis, as inflammation was often observed in the distal 12 cm of the small intestine even when the small intestine was shorter than 36 cm. Thus, the ileum was defined as the distal 12 cm of the small intestine regardless of its length. Ilea were flushed with ice-cold PBS and Swiss-rolled before being fixed in 10% neutral-buffered formalin (10% NBF). Colons were processed as described for the ilea. Lungs, after being lavaged, were inflation-fixed with 10% NBF under a constant pressure. Tissues were then processed, paraffin embedded, and cut into 4 μm thick sections onto Superfrost slides in preparation for staining. Slides were stained either with haematoxylin and eosin (H&E) for general inflammation and tissue histopathology, or Masson’s trichrome or picrosirius red for fibrosis. Slides were scanned using the Leica Aperio AT2 Turbo Brightfield slide scanner (Leica Biosystems, Mount Waverley, VIC, Australia) and analysed using the Aperio ImageScope digital slide viewer (v12.4.0.5043, Leica Biosystems).

Ileitis was graded using the parameters indicated in Supplemental Table S1 [26]. Colitis was graded as described [43]. Muscularis thickness was quantified by tracing either side of the muscularis and measuring 10 lines between each side. This was repeated across 3 regions of the intestine to get the average of 30 measurements. Regions of transmural inflammation were avoided due to poor definition between the muscularis and the lamina propria (LP), and regions where the intestine was incorrectly orientated were also excluded. Alveolar airspace size was quantified using the mean linear intercept (MLI) method [39]. Briefly, a total of 20 equal length lines were drawn over 10 representative images of lung tissue cross-sections stained with H&E and the number of intercepts with alveolar walls were counted. MLI was the result of dividing the line length with the average number of intercepts per line. Lung and intestinal fibrosis were quantified on Masson’s trichrome or picrosirius red stained tissue sections using one of two positive pixel count algorithms depending on the stain used. The parameters used for each are indicated in Supplemental Table S2. Ileal fibrosis was quantified per high power field (hpf) and averaged across three representative images. The parameters used for analysis of lung consolidation are indicated in Supplemental Table S2.

### Single-cell preparation

Single-cell suspensions of the intraepithelial lymphocyte (IEL) populations of the ileum were prepared by flushing the intestines with ice-cold PBS, cutting into pieces ~ 1 cm long and collecting by centrifugation. The intestinal pieces were then finely cut, suspended in Hanks’ Balanced Salt Solution with 10% FCS, 15 mM HEPES and 5 mM EDTA, and agitated at 100 rpm for 40 min at 37 °C, then strained and the suspension collected. The undissociated tissue was then used to isolate LP cells by further processing using the gentleMACS Octo Dissociator with Heaters (Miltenyi Biotec, Macquarie Park, NSW, Australia) in 50 µg/mL Liberase (Merck, Bayswater, VIC, Australia) and 40 µg/mL DNase I (Sigma-Aldrich, St. Louis, MO, USA) using the 37C_m_LPDK_1 preinstalled program, before being washed and strained. Similarly, single-cell suspensions of whole lung tissue that had been lavaged were obtained using the gentleMACS Octo Dissociator with Heaters (Miltenyi Biotec) in 50 µg/mL Liberase (Merck) and 40 µg/mL DNase I (Sigma-Aldrich) using the 37C_m_LDK_1 preinstalled program before performing red blood cell lysis, washing, straining, and a debris removal step (Miltenyi Biotec). Both the LP and lung were processed using the gentleMACS for consistency and to allow both the lungs and intestines to be assessed from the same mouse.

### Flow cytometric analyses

Single-cell suspensions of spleen, IEL, LP, and whole lung were analysed on an LSR-Fortessa X-20 (BD Biosciences, Franklin Lakes, NJ, USA) using the following monoclonal antibodies and Fluorogold viability dye (Santa Cruz Biotechnology, Dallas, TX, USA): BV421-anti-Siglec-F (E50-2440, BD Biosciences), APC-anti-CD4 (RM4-5, BD Bioscience), Alexa Fluor 700-anti-CD45 (30-F11, Invitrogen, Waltham, MA, USA), APC-e709-anti-CD11b (M1/70, eBioscience, San Diego, CA, USA), APC-e780-anti-CD8α (53 − 6.7, eBioscience), PE-anti-Ly6G (1A8, BD Bioscience), PE-anti-γδ TCR (GL3, Biolegend, San Diego, CA, USA), PE Cy7-anti-CD11c (N418, eBioscience), anti-CD3e-biotin (145-2C11, Invitrogen), streptavidin-PE Cy7 (eBioscience). FlowJo software (Windows v10, FlowJo LLC, Ashland, OR, USA) was used to analyse acquired data. Absolute numbers of cells were calculated from cell counts acquired on a Coulter Counter (Beckman Coulter, Mount Waverley, VIC, Australia) and proportions determined by flow cytometry. The cell surface markers used to delineate the key immune cell subsets assessed in this study are defined in Supplemental Table S3.

### Cytokine analysis of BALF and serum

Cytokine levels were analysed using the Bio-Plex Pro™ Mouse Cytokine 23-plex Assay (Bio-Rad, Hercules, CA, USA) following the manufacturer’s instructions. Analyses were performed using the Bio-Plex^®^ 200 (Bio-Rad).

### Characterisation of bacterial communities in the gut of mutant mice

Male and female littermate SHIP-1^+/-^ and SHIP-1^-/-^ mice were housed together until 12-weeks of age whereupon faecal samples were collected. The mice were then euthanised, intestines collected and a diagnosis of ileitis made based on macroscopic and histologic evidence of ileal inflammation. Eight faecal samples from SHIP-1^+/-^ mice and SHIP-1^-/-^ with and without ileitis were then further processed. Bacterial DNA was purified from faeces using the FastDNA™ Spin Kit for Feces (MP Biomedicals, Santa Ana, CA, USA). Ribosomal 16 S next generation sequencing was undertaken on an Illumina MiSeq platform using Paired End v3 2 × 300 chemistry at MicroMon Genomics (Monash University, Clayton, VIC, Australia).

### Amplicon sequencing data analysis

Raw sequencing data were processed using the microbiome-dada2 workflow (https://github.com/respiratory-immunology-lab/microbiome-dada2) using the dada2 R package (version 1.22.0). Briefly, primers and adapters were removed using cutadapt (version 2.1), reads were then filtered and trimmed, sequencing error models were created, sequences were dereplicated, amplicon sequence variants (ASVs) were inferred, paired-end reads were merged, and chimeric sequences removed. Taxonomy assignment was performed using the SILVA database train set and species assignment dataset (version 138.1) to ensure precise sequence matching. A phylogenetic tree of ASV sequences was generated through various alignment processes using the DECIPHER R package (version 2.22.0), which was then followed by the construction of a neighbour-joining tree utilising the phangor R package (version 2.8.1). This tree was used for further maximum likelihood tree analysis, using the Generalised time-reversible model with Gamma rate variation (GTR + G + I). ASV tables along with their corresponding trees and taxonomies were imported into a phyloseq object from the phyloseq R package (version 1.44.0). ASVs exhibiting less than 20% prevalence or those not classified at the phylum level were filtered out. Alpha diversity measures such as the Shannon and Chao1 indexes were determined using the estimate_richness function within phyloseq. ASV counts were normalised using Cumulative Sum Scaling (CSS) by employing the calcNormFactors function of the MetagenomeSeq R package (version 1.42.0). Principal Coordinate Analysis (PCoA) was performed using the weighted Unifrac distance matrix of the normalised ASV counts. ANOSIM (ANalysis Of Similarities) was used to evaluate the effect of genotype and ileitis on overall microbiota composition (weighted Unifrac distance matrix) with the function ‘’Ileitis + Genotype’’ using the adonis2 function from the vegan R package (version 2.6.4). Differential abundance testing of ASVs was performed using the ‘’metagenomeSeq’’ method available from microeco R package (version 0.20.0) with FDR-adjusted *P*-values.

### Statistics

Statistical analyses were performed using GraphPad Prism V.9.3.1 (GraphPad, La Jolla, CA, USA). Data were analysed by nonparametric Kruskal-Wallis test (nonparametric ANOVA) for comparison of three or more groups. Data points are presented as median ± interquartile range (IQR) and a *P*-value < 0.05 was considered statistically significant.

## Results

### Ileitis development in SHIP-1-/- mice is dependent on housing environment

Two previous independent studies of SHIP-1^−/−^ mice have reported that they develop intestinal inflammation by 8 weeks of age with almost complete penetrance [26, 27]. Since environmental factors are known to contribute to the pathogenesis of CD in patients [44], as well as DSS-induced colitis in mice [45], this was assessed in SHIP-1^−/−^ mice that were bred in a high-barrier SPF facility before being shipped at weaning to one of two facilities of differing health status: a higher-barrier SPF facility and a lower-barrier SPF facility. Mice were moved into one of the two facilities at weaning since this is known to be a key time point when major changes in the intestinal microbiota and immune responses occur [46]. Neither facility induced full disease penetrance of ileitis by 12 weeks of age, and it was observed that the higher-barrier SPF facility had a profoundly protective effect on ileitis development, with no ileitis detected during the period of observation (Fig. 1A). These data indicate that genetics alone is insufficient to induce CD-like ileitis in the SHIP-1-deficient model.

Incomplete penetrance of ileitis afforded the opportunity to dissect the immunological processes of ileitis from the effects of SHIP-1-deficiency alone. SHIP-1^−/−^ mice with ileitis, which was diagnosed at necropsy, were observed to have similar body weights to ileitis-free SHIP-1^−/−^ mice by 12 weeks of age (Fig. 1B), and while their spleen weight was not significantly different (Fig. 1C), they exhibited a significantly increased spleen weight to body weight ratio (Fig. 1D) and an increased shortening of the small intestine (Fig. 1E). The increase in spleen weight is potentially attributed to increased numbers of myeloid cells, including neutrophils and eosinophils (Fig. 1F and G), which likely result from enhanced extramedullary haematopoiesis, a feature of SHIP-1 deficiency [37, 47]. This demonstrates that SHIP-1^−/−^ mice with ileitis have a more severe inflammatory phenotype, although it is unclear whether this is the result or cause of ileitis.

### SHIP-1-/- mice without ileitis have normal ileal structure and immune cell compartment

Ileitis in SHIP-1^−/−^ mice is characterised by polymorphonuclear (PMN) infiltration, granuloma formation, transmural inflammation, fibrosis development, loss of normal villus and crypt architecture, and muscularis thickening [26, 27]. We compared the ilea of 12-week-old SHIP-1^−/−^ mice with and without macroscopically obvious ileitis using histological and flow cytometric analyses to determine if there were any characteristic differences. SHIP-1^−/−^ mice that did not develop overt ileitis had ileal architecture reminiscent of control mice without abnormal immune cell infiltrates or fibrosis development (Fig. 1H and I). Furthermore, SHIP-1^−/−^ mice without overt macroscopic ileitis had low ileitis grades, normal muscularis thickness, and normal levels of intestinal collagen (fibrosis) whereas SHIP-1^−/−^ mice with ileitis had high ileitis grades, a significantly thickened muscularis, and evident fibrosis (Fig. 1I and K).

**Fig. 2:**
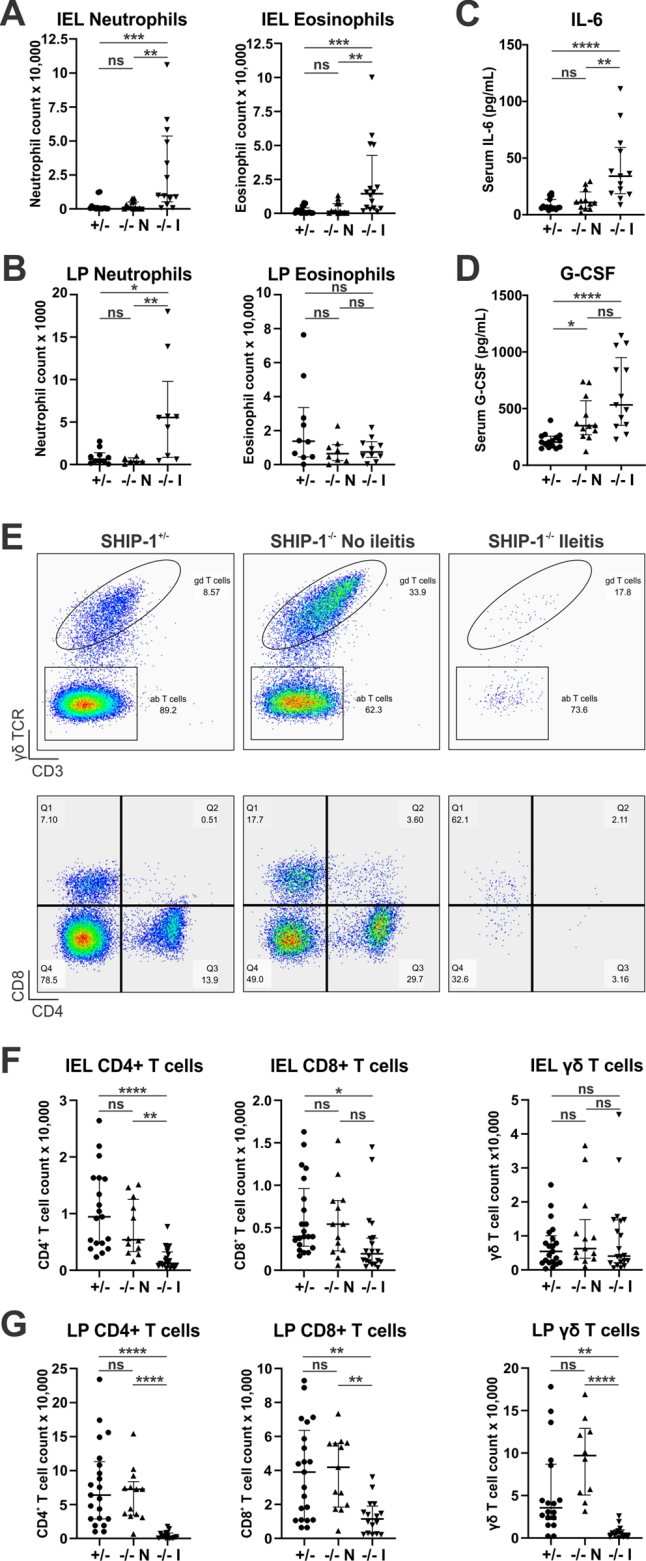
SHIP-1^−/−^ mice lacking ileitis have ileal leukocyte populations comparable to control mice. Ileal tissue from low barrier facility housed 12-week-old SHIP-1^+/−^ mice (+/-), SHIP-1^−/−^ mice without ileitis (-/- N), and SHIP-1^−/−^ mice with ileitis (-/- I) was analysed by flow cytometry. Total numbers of neutrophils (CD45^+^CD11c^−^CD11b^+^Siglec-F^−^Gr-1^+^) and eosinophils (CD45^+^CD11c^−^CD11b^+^Siglec-F^+^Gr-1^−^) in the **A** IEL and **B** LP tissue fractions as determined by cell counts and flow cytometry. Concentration (pg/mL) of **C** IL-6 and **D** G-CSF in serum. **E** Representative flow cytometry pseudocolour plots depicting γδ T cells (top panels) and CD4^+^ and CD8^+^ T cells (bottom panels) in the LP, after gating on live CD45^+^ cells. Note the low number of events in samples of SHIP-1^−/−^ mice with ileitis are due to reduced T cell numbers in these samples. Total numbers of CD4^+^, CD8^+^ and γδ T cells in the **F** IEL and **G** LP tissue fractions. Data is presented as median ± IQR. ns = not significant; * *P* < 0.05; ** *P* < 0.01; *** *P* < 0.001; **** *P* < 0.0001 by Kruskal-Wallis non-parametric ANOVA test comparing SHIP-1^+/−^, SHIP-1^−/−^ without ileitis and SHIP-1^−/−^ with ileitis (**A-D**,** F-G**)

In addition to the lack of histological changes to the ileum of SHIP-1^−/−^ mice without overt ileitis, the immune cell composition of the ileum was comparable to that of control mice (Fig. 2). While granulocyte infiltration, which included CD45^+^CD11b^+^Ly6G^+^ neutrophils and CD45^+^CD11b^+^Siglec-F^+^ eosinophils, was a feature of SHIP-1^−/−^ mice with ileitis, this was not observed in the IEL or LP of SHIP-1^−/−^ mice without ileitis (Fig. 2A and B). The inflammation in the ilea coincided with a significant increase in concentration of IL-6 protein in the serum (Fig. 2C), and while G-CSF levels were elevated, this was also observed in SHIP-1^−/−^ mice without ileitis (Fig. 2D). Importantly, both of these proteins regulate granulopoiesis [48], and both have been shown to contribute to systemic inflammation and myeloid cell production in SHIP-1^−/−^ mice [39, 49]. Other differences in circulating cytokines were also noted including increases in IL-5, CCL3 and CCL4 that were not seen in SHIP-1^−/−^ without ileitis (Supplemental Fig. S1). In addition, SHIP-1^−/−^ mice exhibited increases in serum IL-12(p40) and CXCL1, and a reduction in IL-12(p70) and CCL5 that were independent of ileitis development (Supplemental Fig. S1). Interestingly, while there was a reduction of CD4^+^ and CD8^+^ T cells in the IEL and LP, and γδ T cells in the LP of the inflamed ilea, this was not observed in the uninflamed ileum of SHIP-1^−/−^ mice (Fig. 2E-G). However, there was a mild but non-significant increase in γδ T cells in the LP of SHIP-1^−/−^ mice without ileitis compared to both SHIP-1^−/−^ mice with ileitis and SHIP-1^+/−^ controls, suggesting that γδ T cells may expand prior to ileitis onset potentially to regulate intestinal immune responses that could lead to ileitis (Fig. 2G). These data indicate that ileitis does not develop in all adult SHIP-1^−/−^ mice as previously thought [26, 27], and shows that environment plays a key role in susceptibility.

### Ileitis in SHIP-1−/− mice correlates with loss of rare Bifidobacterium ASVs

**Fig. 3:**
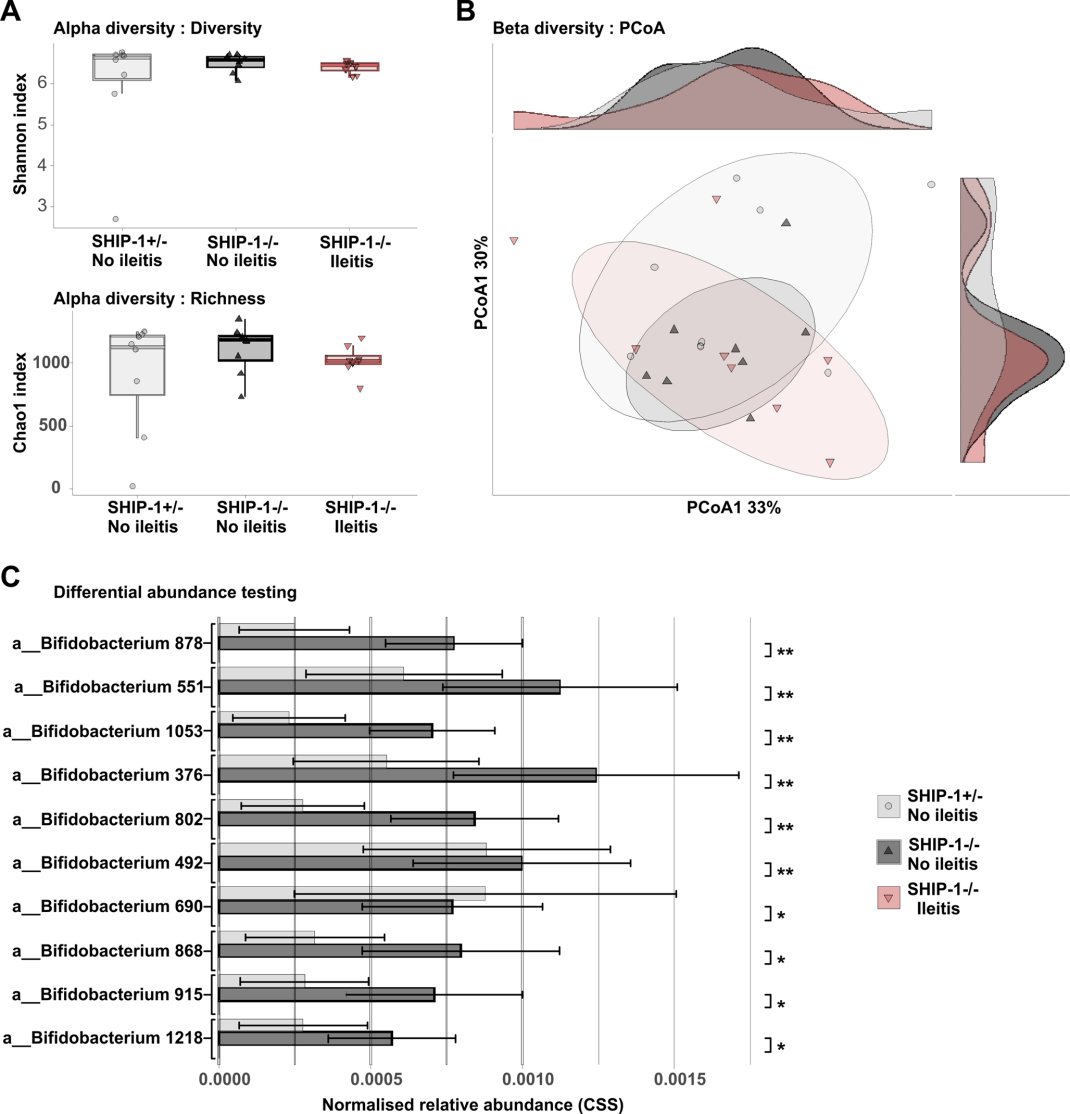
Rare *Bifidobacterium* ASVs are underrepresented in the gut of SHIP-1^−/−^ mice with ileitis. Faecal samples from low barrier facility housed 12-week-old littermate SHIP-1^+/−^ mice, SHIP-1^−/−^ mice without ileitis, and SHIP-1^−/−^ mice with ileitis were processed (*n* = 8 samples of each), subjected to 16 S rRNA sequencing, and data was analysed for **A** Diversity (Shannon index) and Richness (Chao1 index); **B** Principal Coordinate Analysis (PCoA) and corresponding density plots across the groups based on weighted Unifrac distance of the CSS normalised dataset; and, **C** Differential abundance testing using a Zero-inflated Gaussian mixture model (metagenomeSeq) showing CSS-normalised relative abundance per group of significant *Bifidobacterium* ASVs. Data is presented as median *±* SE with statistics representing the *FDR*-corrected *P*-value; * FDR < 0.05; ** FDR < 0.01 between SHIP-1^−/−^ mice with and without ileitis

To determine if gut disease in SHIP-1^−/−^ mice influenced the bacterial microbiome, 16 S ribosomal RNA sequencing was used to characterise and compare the microbial composition of faecal samples from 12-week-old SHIP-1^−/−^ mice with and without ileitis using matched SHIP-1^+/−^ as controls. Comparative analysis revealed no significant variation in microbial diversity or richness among the groups (Fig. 3A). Likewise, Principal Coordinate Analysis (PCoA) demonstrated no changes in the overall composition of the microbial communities (ANOSIM, *R*^2^ = 6% and 5%, *P*-value = 0.2 and 0.15 for ileitis and genotype, respectively) (Fig. 3B). However, low abundance *Bifidobacterium* ASVs were largely absent in SHIP-1^−/−^ mice with ileitis, with the top ten being significantly different, as shown by differential abundance testing (Fig. 3C). Of the 25 total *Bifidobacterium* ASVs present in the dataset, only three were detected in SHIP-1^−/−^ mice with ileitis (Supplemental Fig. S2). These data suggest that *Bifidobacterium* strains may help to protect the gut of SHIP-1^−/−^ mice from inflammation.

### SHIP-1-deficiency promotes lung disease irrespective of ileitis development

**Fig. 4:**
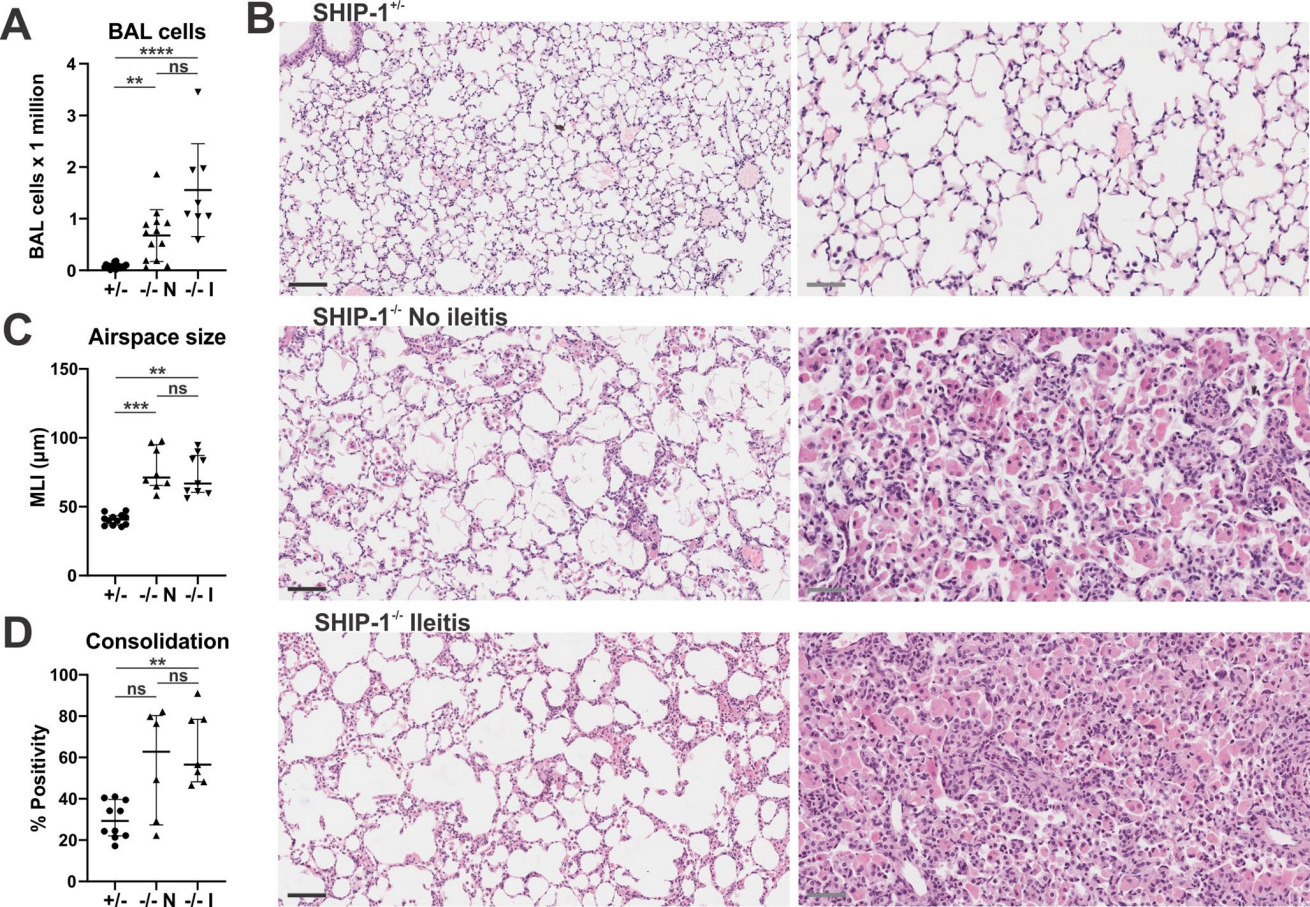
SHIP-1^−/−^ mice develop chronic lung inflammation regardless of ileitis penetrance. The lung tissue of low barrier facility housed 12-week-old SHIP-1^+/−^ mice (+/-), SHIP-1^−/−^ mice without ileitis (-/- N), and SHIP-1^−/−^ mice with ileitis (-/- I) was analysed by cytology and histopathology. **A** BAL cell counts of indicated mice by haemocytometer. **B** Representative images of sections of inflation-fixed lungs stained with H&E depicting regions with less consolidation (left panels) and areas that are heavily consolidated (right panels) for SHIP-1^−/−^ mice. Magnification = 20x (left panels) and 40x (right panels). Scale bars = 100 μm (left panels) and 50 μm (right panels). **C** Airspace size quantified by mean linear intercept (MLI) (µm) in non-consolidated regions of the lung. **D** Consolidation of lung tissue quantified as percentage of tissue compared to airspace in whole lung section. Data is presented as median ± IQR. ns = not significant; ** *P* < 0.01; *** *P* < 0.001; **** *P* < 0.0001 by Kruskal-Wallis non-parametric ANOVA test comparing SHIP-1^+/−^, SHIP-1^−/−^ without ileitis and SHIP-1^−/−^ with ileitis

SHIP-1-deficiency leads to inflammatory lung disease characterised by alternatively-activated alveolar macrophages, pulmonary fibrosis, and the deposition of chitinase crystals within the lungs [36, 50]. Given that ileitis in SHIP-1^−/−^ mice developed with incomplete penetrance, the impact of ileitis upon lung inflammation was then assessed by histopathology and by determining immune cell infiltration into alveolar airspaces. As has been reported previously, SHIP-1^−/−^ mice had increased BAL cell counts compared to control mice [36]; and while this appeared further elevated in SHIP-1^−/−^ mice with ileitis, the differences were not significant (Fig. 4A). Enlargement of alveoli reminiscent of emphysema is a known feature of SHIP-1^−/−^ mice [39, 51]; however, no significant difference in MLI between SHIP-1^−/−^ mice with and without ileitis was observed (Fig. 4B and C), indicating that emphysema was not worsened in the presence of ileitis. Consolidation of the lung tissue, characterised by an increase in immune cells both in the lung tissue and airspaces, is apparent in SHIP-1^−/−^ mice, and whilst this consolidation appears more densely packed in SHIP-1^−/−^ mice with ileitis, no significant difference was noted when consolidation was quantified as a percentage of positive tissue compared to airspaces in the whole lung (Fig. 4B and D). Altogether, these data suggest that the presence of ileitis in SHIP-1^−/−^ mice does not influence inflammation in BAL and does not manifest as a significant difference in lung pathology.

### Ileitis in SHIP-1-/- mice promotes granulocytic lung inflammation

**Fig. 5:**
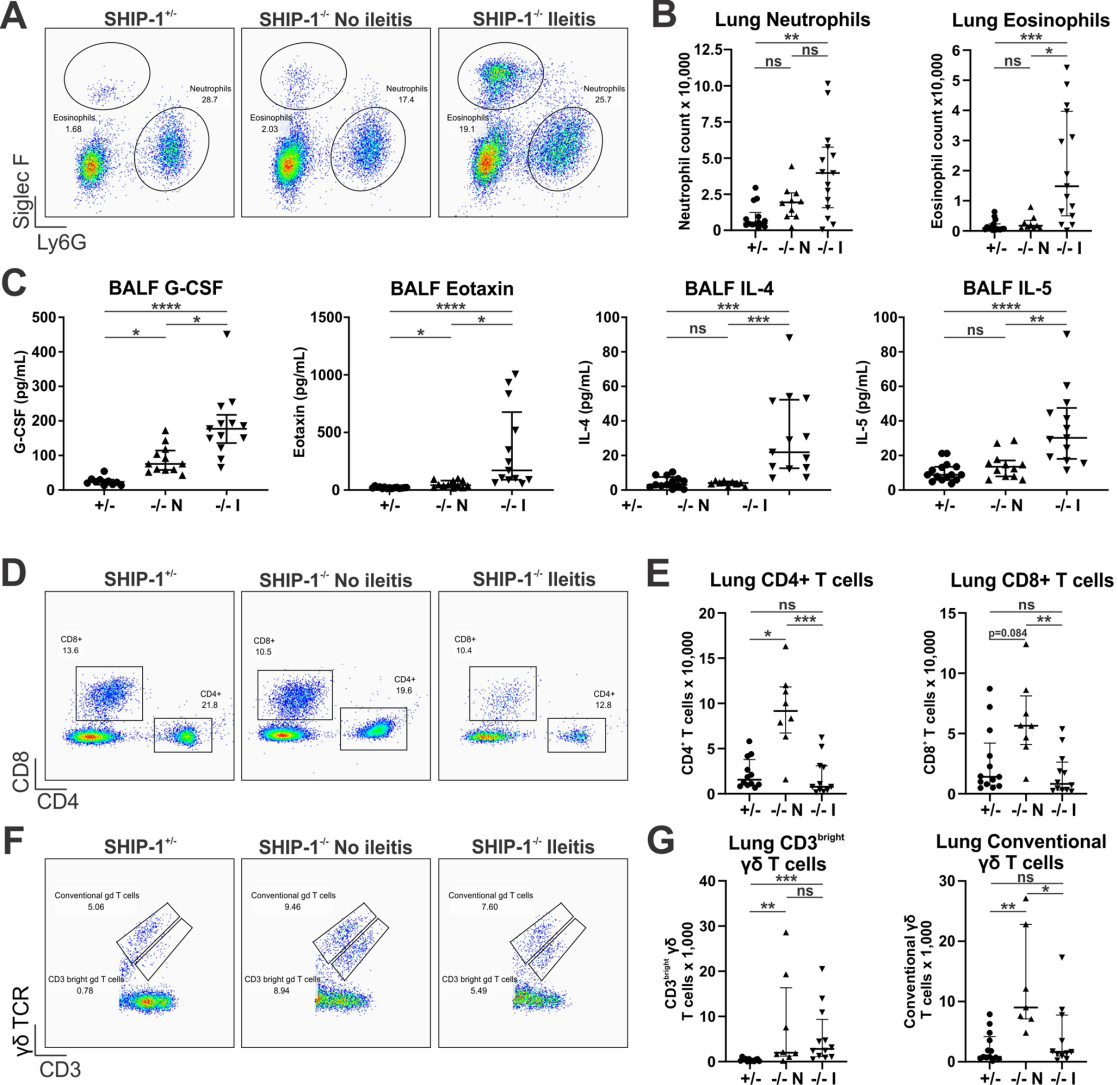
SHIP-1^−/−^ mice develop chronic lung inflammation with a distinct immunological signature that correlates with ileitis. The lung tissue of low barrier facility housed 12-week-old SHIP-1^+/−^ mice (+/-), SHIP-1^−/−^ mice without ileitis (-/- N), and SHIP-1^−/−^ mice with ileitis (-/- I) was analysed by flow cytometry. **A** Representative flow cytometry pseudocolour plots of lung tissue neutrophils (Ly6G^+^Siglec-F^−^) and eosinophils (Ly6G^−^Siglec-F^+^), after gating on live CD45^+^CD11c^−^CD11b^+^ cells. **B** Total number of neutrophils and eosinophils in the left lung lobe by cell counts and flow cytometry. **C** Concentration of cytokines in BALF. **D** Representative flow cytometry pseudocolour plots of lung tissue CD4^+^ and CD8^+^ T cells gated on CD45^+^CD3^+^γδTCR^−^ cells. **E** Total number of CD4^+^ and CD8^+^ T cells in the left lung lobe by cell counts and flow cytometry. **F** Representative flow cytometry pseudocolour plots of lung tissue CD45^+^CD3^+^ γδ T cells. **G** Number of CD3^bright^ and conventional γδ T cells in the left lung lobe by cell counts and flow cytometry. Data is shown as median ± IQR. ns = not significant; * *P* < 0.05; ** *P* < 0.01; *** *P* < 0.001; **** *P* < 0.0001 by Kruskal-Wallis non-parametric ANOVA test comparing SHIP-1^+/−^, SHIP-1^−/−^ without ileitis and SHIP-1^−/−^ with ileitis

Due to the mildly increased inflammation in the airspaces of SHIP-1^−/−^ mice that harboured ileitis, flow cytometry was next used to examine inflammation in whole lung tissue. Interestingly, the lung tissue of SHIP-1^−/−^ mice with ileitis exhibited a significant expansion of both neutrophils and eosinophils that was not present in SHIP-1^−/−^ mice lacking ileitis (Fig. 5A and B). The expansion of neutrophils in the BALF of SHIP-1^−/−^ mice with ileitis coincided with a significant increase in the key neutrophil regulator G-CSF, while the expansion of eosinophils correlated with significant increases eosinophil regulators in BALF including eotaxin, IL-4 and IL-5 (Fig. 5C). Other factors that were only increased in the BALF of SHIP-1^−/−^ mice with ileitis were IL-33 and TNFα, while IL-6, CCL2, and CCL4 were more significantly increased in the lungs of SHIP-1^−/−^ mice with ileitis (Supplemental Fig. S3).

In the lungs of SHIP-1^−/−^ mice without ileitis there was an expansion of CD4^+^ and CD8^+^ T cells compared to controls, although this was not seen in SHIP-1^−/−^ mice with ileitis (Fig. 5D and E). Despite this, there was a decrease in T cell cytokines including interferon-γ and IL-2 in the BALF of both cohorts of SHIP-1^−/−^ mice (Supplemental Fig. S3). The γδ T cell compartment was also altered in the lungs of SHIP-1^−/−^ mice with an increase in CD3^bright^ γδ T cells, which have been described as IL-17-producing [52]. This was noted in all SHIIP-1^−/−^ mice regardless of gut inflammation, although an expansion of conventional γδ T cells was also observed in SHIP-1^−/−^ mice that did not develop ileitis (Fig. 5F and G). These data show an association between pulmonary granulocytophilia and presence of ileitis in SHIP-1^−/−^ mice as well as an association between conventional T cell expansion in the lungs and a lack of ileitis.

### Deletion of G-CSF in SHIP-1-/- mice prevents the development of ileitis

**Fig. 6:**
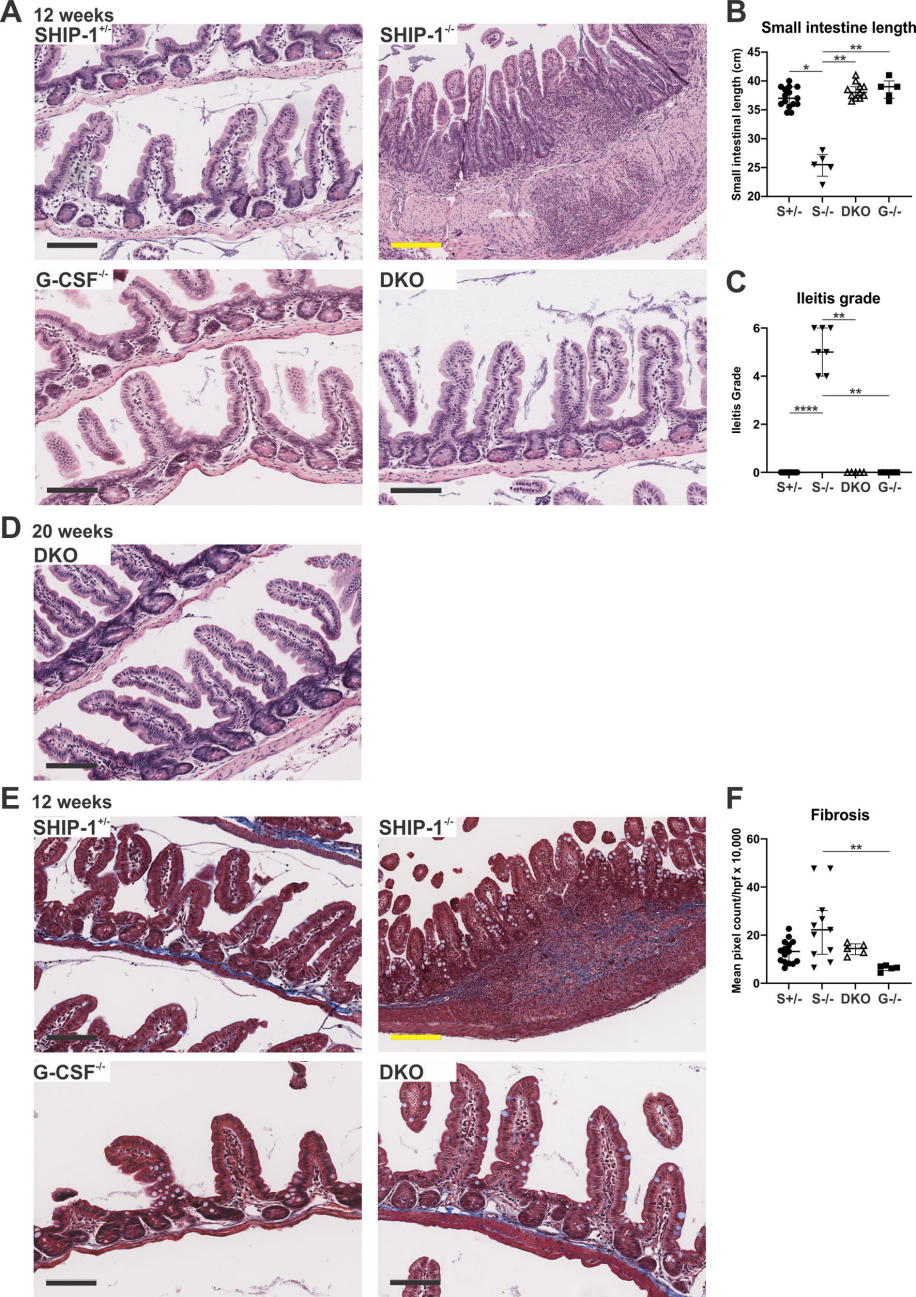
G-CSF-deficiency in SHIP-1^-/-^ mice is protective against ileitis development. **A** Representative H&E-stained Swiss-rolled ileal sections from the indicated low barrier facility housed 12-week-old mice. Scale bars = 100 μm for SHIP-1^+/−^, G-CSF^−/−^ and DKO mice, and 200 μm for SHIP-1^−/−^ mice. **B** Small intestinal length (cm) of the indicated 12-week-old mice (S+/- = SHIP-1^+/−^, S-/- = SHIP-1^−/−^ mice with ileitis, DKO = SHIP-1^−/−^G-CSF^−/−^ G-/- = G-CSF^−/−^). **C** Histopathological grading of ileitis. **D** Representative section of Swiss-rolled ilea stained with H&E from 20-week-old DKO mice. **E** Representative Masson’s Trichrome-stained sections of Swiss-rolled ilea. Scale bars = 100 μm for SHIP-1^+/−^, G-CSF^−/−^ and DKO mice, and 200 μm for SHIP-1^−/−^ mice. **F** Quantification of Masson’s Trichrome staining represented as average total positive pixels per high power field (hpf). Data is presented as median ± IQR. * *P* < 0.05; ** *P* < 0.01; **** *P* < 0.0001 by Kruskal-Wallis non-parametric ANOVA test comparing S+/-, S-/- with ileitis, DKO and G-/- mice. Non-significant differences are unmarked

Previous work reported that deletion of G-CSF ameliorates lung pathology and associated comorbidities in SHIP-1^−/−^ mice [39]; however, the ileitis phenotype was not assessed. To determine the contribution of G-CSF to intestinal pathology, we examined the ilea of 12-week-old SHIP-1^−/−^G-CSF^−/−^ double-knockout (DKO) mice and found no evidence of ileitis (Fig. 6). Due to our finding that housing conditions contributed to the intestinal phenotype (Fig. 1A), mice were housed in a lower-barrier animal facility from weaning where ileitis had been observed in 60% of SHIP-1^−/−^ mice. Nonetheless, we were unable to promote intestinal inflammation in DKO mice even in the face of a higher pathogen load (Fig. 6). The ilea of 12-week-old DKO mice appeared normal with no evidence of immune cell infiltration thus preventing the shortening of the small intestine and resulting pathology seen in SHIP-1^−/−^ mice harbouring ileitis (Fig. 6A-C). To determine if G-CSF deficiency slowed the development of ileitis or provided complete protection, DKO mice were aged to 20-weeks in the lower barrier facility. Similarly, aged DKO mice showed no evidence of ileitis suggesting G-CSF-deficiency provided prolonged protection against intestinal inflammation (Fig. 6D). Ileitis in SHIP-1^−/−^ mice is characterised by arginase-1-dependent fibrosis [27]. To determine whether G-CSF-deficiency protected against fibrosis development, Swiss-rolled ilea were stained with Masson’s Trichrome, and staining was quantified as positive pixel counts. DKO mice did not develop the characteristic increase in fibrosis observed in SHIP-1^−/−^ mice harbouring ileitis (Fig. 6E-F). Together, these data show that G-CSF-deficiency in the SHIP-1^−/−^ model of spontaneous intestinal inflammation is sufficient to prevent ileitis development.

**Fig. 7:**
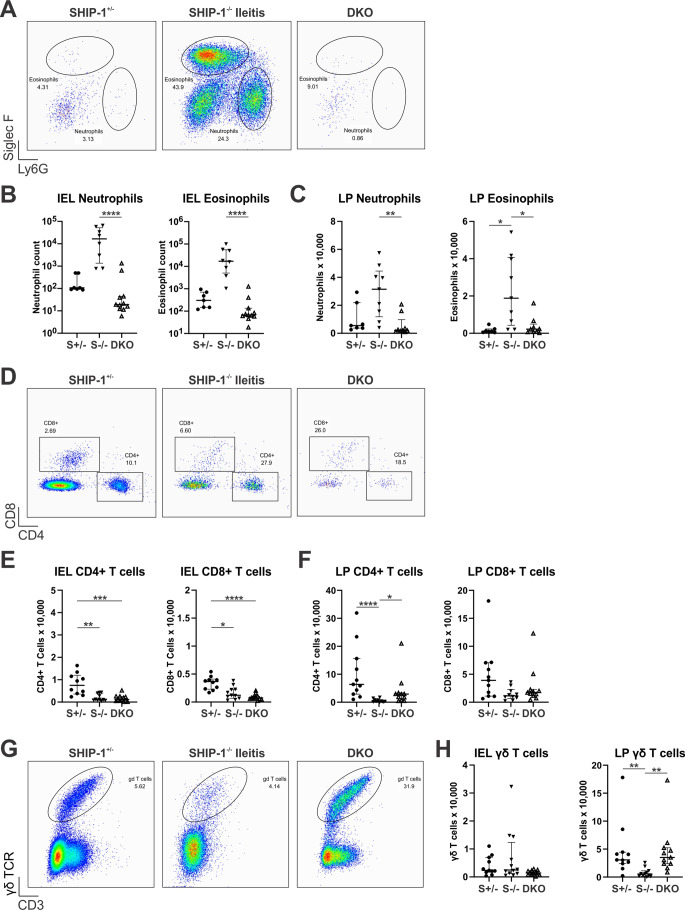
G-CSF-deficiency in SHIP-1^-/-^ mice protects against ileitis and granulocyte infiltration into the ileum. Ileal tissue from low barrier facility housed 12-week-old SHIP-1^+/−^ mice (S+/-), SHIP-1^−/−^ mice with ileitis (S-/-) and SHIP-1^−/−^G-CSF^−/−^ mice (DKO) was analysed by flow cytometry. **A** Representative flow cytometry pseudocolour plots of IEL fraction depicting neutrophils (CD45^+^CD11c^−^CD11b^+^Siglec-F^−^Gr-1^+^) and eosinophils (CD45^+^CD11c^−^CD11b^+^Siglec-F^+^Gr-1^−^). Note the low number of events in samples of SHIP-1^+/−^ mice and SHIP-1^−/−^ mice without ileitis are due to a lack of ileal inflammation. **B** Total numbers of neutrophils and eosinophils in IEL as determined by cell counts and flow cytometry shown in panel A. **C** Total numbers of neutrophils and eosinophils in LP as determined by cell counts and flow cytometry. **D** Representative flow cytometry pseudocolour plots of IEL depicting CD4^+^ (CD45^+^CD3^+^γδTCR^−^CD4^+^) and CD8^+^ (CD45^+^CD3^+^γδTCR^−^CD8^+^) T cells. Note the low number of events in samples of SHIP-1^−/−^ mice with ileitis are due to reduced T cell numbers in these samples. Total numbers of CD4^+^ and CD8^+^ T cells in **E** IEL and **F** LP as determined by cell counts and flow cytometry. **G** Representative flow cytometry pseudocolour plots depicting γδ T cells (CD45^+^CD3^+^γδTCR^+^) gated on lymphocytes (based on FSC-A and SSC-A). **H** Total numbers of γδ T cells in the IEL and LP of mice as determined by cell counts and flow cytometry. Data is presented as median ± IQR. ns = not significant; * *P* < 0.05; ** *P* < 0.01; *** *P* < 0.001; **** *P* < 0.0001 by Kruskal-Wallis non-parametric ANOVA test comparing S+/-, S-/- with ileitis and DKO mice. Non-significant differences are unmarked

To determine whether G-CSF deficiency in SHIP-1^−/−^ mice had an impact upon leukocyte populations within the gut, we assessed the ileum via flow cytometry. In keeping with their lack of ileitis phenotype, the ileal tissue of DKO mice looked comparable to control mice with similar numbers of neutrophils and eosinophils, and this contrasted with inflamed tissue from SHIP-1^−/−^ mice harbouring ileitis, where increased numbers of these myeloid cells were found (Fig. 7A-C). With regards to lymphocyte subsets, DKO mice had a profile similar to SHIP-1^−/−^ mice, exhibiting a lymphopenia phenotype with reductions in CD4^+^ and CD8^+^ T cells (Fig. 7D-F). Furthermore, DKO mice had similar numbers of γδ T cells in the IEL and elevated numbers in LP when compared to SHIP-1^−/−^ mice (Fig. 7G-H). This suggests that T cell lymphopenia is not the key driving factor of ileitis development in SHIP-1^−/−^ mice but may instead depend on an expansion of PMNs, although γδ T cells may play a protective role.

## Discussion

This study shows that Crohn’s-like ileitis is not a fully penetrant phenotype of SHIP-1^−/−^ mice, which is distinct from previous studies that reported disease in almost all mice [26, 27]. Strain differences may be a factor; however, while all were independently created, they were made by targeting the first coding exon of *Inpp5d* and disruption of SHIP-1 protein production [37, 38, 53]. Genetic variation might also contribute as the mice used herein were on a C57BL/6 background [36]; however, while one group examined mixed genetic background SHIP-1^−/−^ mice [27], those studied by the second group were similarly backcrossed to C57BL/6 [26]. A credible explanation for phenotypic differences is the housing environment and associated pathogen load. Under high barrier SPF conditions, no ileitis was recorded in SHIP-1^−/−^ mice, while in a lower-barrier facility, approx. 60% of mice developed ileitis by 12 weeks of age. Thus, genetics alone is insufficient to drive gut inflammation, suggesting that other environmental factors are required to initiate or protect from disease in this model. This is not surprising since housing environment has been shown to impact disease severity in other IBD models including DSS-induced colitis [45]. Moreover, environmental factors play a large role in development of CD. Components such as cigarette smoking, early-life antibiotics, air pollution, and a Westernised lifestyle (increased hygiene, consumption of highly processed, high fat foods) that are associated with disease onset [54], all impact upon the gut microbiota.

Curiously, while there were few global differences in the gut microbiota of SHIP-1^−/−^ mice with and without ileitis, mice harbouring ileitis mostly lacked low abundance *Bifidobacterium* ASVs. In our dataset, only 3/25 *Bifidobacterium* ASVs were detectable in SHIP-1^−/−^ mice with ileitis, whereas all were found in control mice or SHIP-1^−/−^ mice lacking gut inflammation. In humans, *Bifidobacterium* has protective anti-inflammatory properties and is used as a probiotic [55]. *Bifidobacterium* can also protect IL-10^−/−^ mice from developing colitis [56], and it is beneficial in mice with DSS-induced colitis [57–59] and in the SAMP/Yit spontaneous model of Crohn’s-like ileitis [60]. Similarly, our data suggests that *Bifidobacterium* protects SHIP-1^−/−^ mice from gut inflammation; however, it is presently unclear why specific *Bifidobacterium* strains are only absent from SHIP-1^−/−^ mice with ileitis. The mice used in this study were littermates housed together in groups and although mice are coprophagic and likely share microbes, there was no correlation between development of gut inflammation and cage representation, as SHIP-1^−/−^ mice with or without ileitis were often housed together. How *Bifidobacterium* species protects SHIP-1^−/−^ mice is uncertain but its use as a probiotic in this model may prevent the development of intestinal inflammation, as seen in other disease models [56–60]. A potential limitation of these studies is the use of faeces as a surrogate for microbial representation at the ileum; however, this is more relevant clinically, and despite this, the loss of a key species identified in faeces correlated with a striking difference in disease incidence. It is of note that the T cell compartment of SHIP-1^−/−^ mice with ileitis was markedly altered, with significant reductions in IEL and LP T cells, which were unchanged in the uninflamed gut of SHIP-1^−/−^ mice. In addition, LP γδ T cells were also significantly reduced, while they were significantly increased in the uninflamed gut of SHIP-1^−/−^ mice. *Bifidobacterium* are known to induce intestinal Th17 cells [61], enhance Treg mitochondrial activity, promoting IL-10 production and protecting against colitis [62], and are positively correlated with upregulation of γδ T cells in the colonic LP in a DSS-colitis model [63]. Thus, loss of *Bifidobacterium* species in the gut of SHIP-1^−/−^ mice may predispose to inflammation due to T cell compartment changes. Studies on younger mice and the emergence of these phenotypes may shed further light.

Partial penetrance of ileitis in SHIP-1^−/−^ mice allowed us to examine if the intestinal environment influenced lung inflammation, since all SHIP-1^−/−^ mice develop COPD-like lung disease [36, 40]. The lungs of SHIP-1^−/−^ mice with ileitis showed an enhanced type 2 signature compared to those without gut disease. Consistent with this, eosinophilia in the lungs of patients with CD but without overt lung disease has been reported [64]. Additionally, it has been recently shown that the commensal gut protozoa *Tritrichomonas musculis* can act as a pathobiont and induce lung eosinophilia and predispose mice to asthma [65]. The fact that protective *Bifidobacterium* strains were poorly represented in the gut microbiome of SHIP-1^−/−^ mice that developed ileitis, suggests that this may somehow predispose to lung eosinophilia. The lungs of SHIP-1^−/−^ mice with ileitis also had a strong neutrophil signature. We have recently shown that the development of severe colitis is linked with expansion of neutrophils and alveolar macrophages in the lung as well as increases in neutrophil genes and lung tissue damage [66], demonstrating a link between the two tissue sites in disease. It seems likely that there is a relationship between the mixed granulocytic signature in the lungs of SHIP-1^−/−^ mice that co-presents exclusively with inflamed ilea that also expresses the same signature. Given that not all SHIP-1^−/−^ mice develop ileitis, it is possible that this signature arises in the lung, with inflammatory cell spillover leading to granulocytes trafficking to the gut. Alternatively, absolute pathogen load at each mucosal site may independently determine local granulocyte responses but more work is required to delineate between these scenarios.

G-CSF-deficiency in SHIP-1^−/−^ mice is known to protect against the development of lung inflammation and extrapulmonary diseases such as right heart hypertrophy, loss of fat reserves, and osteoporosis [39]. We have now shown that it also completely prevented the development of ileitis. While ileal tissue from SHIP-1^−/−^G-CSF^−/−^ mice was protected from infiltrating myeloid cells, the T cell lymphopenia characteristic of SHIP-1^−/−^ ilea was still observed, suggesting that reduced T cell activity does not contribute to ileitis. The protection of SHIP-1^−/−^G-CSF^−/−^ mice from ileitis was unexpected given that mice deficient in the G-CSF receptor are more susceptible to DSS-induced colitis, although this has been attributed to impairment of immunoregulatory macrophages in colon and not neutrophils [67]. Recombinant G-CSF has been suggested as a therapeutic for colitis, showing efficacy in mice [68], as well as in patients with active CD [69]. We have similarly found that G-CSF^−/−^ mice are more susceptible to DSS-induced colitis (unpublished) and speculate that differing mechanisms and location of disease between DSS colitis and Crohn’s-like ileitis in SHIP-1^−/−^ mice may account for the contrasting effects of G-CSF-deficiency on gut inflammation. In fact, SHIP-1^−/−^ mice have been shown to be protected from DSS-induced colitis due to the presence of alternatively-activated macrophages [70], suggesting distinct mechanisms for colitis and ileitis. G-CSF-deficiency in SHIP-1^−/−^ is likely protective against ileitis due to a reduction of neutrophils, which are prominent in the inflamed ileum of SHIP-1^−/−^ mice [26, 27]. SHIP-1 deficiency ordinarily enhances neutrophil survival [38], but when coupled with G-CSF deficiency, the augmentation of neutrophil development, survival, and activation is lost [39]. Furthermore, SHIP-1^−/−^ mice typically exhibit enhanced haematopoietic stem cell proliferation that is skewed towards myelopoiesis [47, 49]; however, this is markedly reduced in SHIP-1^−/−^G-CSF^−/−^ mice [39]. Overall, this suggests that blocking G-CSF might be efficacious for CD therapy. Supporting this, IL-23, a regulator of neutrophil homeostasis, is a proven target with ustekinumab (targeting IL-12/IL-23 p40) now in use in the IBD clinic following successful clinical trials [71].

In conclusion, this study establishes that environmental factors contribute to ileitis development in SHIP-1^−/−^ mice, with *Bifidobacterium* strains identified as being potentially protective against disease. Furthermore, the presence of ileitis impacted the nature of lung inflammation in the model, identifying granulocytes as possible key cell targets of interest in comorbid ileitis and COPD. Additionally, G-CSF was found to be essential for the development of ileitis in SHIP-1^−/−^ mice, suggesting that hyperactive neutrophils or their products drive ileitis, which could in part influence their interaction with the microbiota.

## Electronic supplementary material

Below is the link to the electronic supplementary material.

Supplementary Material 1

## Data Availability

Raw 16 S amplicons sequencing data have been deposited at the National Centre for Biotechnology Information database under the BioProject accession number PRJNA1086166. All other data are available in the main text or the supplementary materials.
