# Supplementary material for: Environmental and inflammatory factors influencing concurrent gut and lung inflammation
Source: Inflamm Res. 2024 Oct 21;73(12):2123–39. doi: 10.1007/s00011-024-01953-x (PMC11632041; doi:10.1007/s00011-024-01953-x)

**Supplementary Information**

**Table S1.** Criteria used to grade ileitis.

| **Grade (0-6)** | **Criteria** |
| --- | --- |
| 0 | No abnormalities |
| 1 | Mild, predominately PMN leukocyte infiltration (<25 PMN/hpf) of the lamina propria. |
| 2 | Moderate, predominantly PMN leukocyte infiltration (>25 PMN/hpf) of the lamina propria. |
| 3 | Marked inflammatory cell infiltration extending below the muscularis mucosa. Architectural distortion of the mucosa and submucosa. Crypt hyperplasia. |
| 4 | Marked infiltration extending into the tunica muscularis. |
| 5 | Marked transmural infiltration. |
| 6 | Marked transmural infiltration extending into the mesentery. |

#### **Table S2.** Positive pixel count algorithm input parameters for disease scoring.

| **Parameter** | **Input parameters to analyse fibrosis in Masson’s trichrome stained images** | **Input parameters to analyse fibrosis in picrosirius red stained images** | **Input parameters for analysis of lung consolidation** |
| --- | --- | --- | --- |
| View Width | 1000 | 1000 | 1000 |
| View Height | 1000 | 1000 | 1000 |
| Overlap Size | 0 | 0 | 0 |
| Image zoom | 1 | 1 | 1 |
| Mark-up Compression Type | Same as processed image | Same as processed image | Same as processed image |
| Compression Quality | 30 | 30 | 30 |
| Classifier Neighbourhood | 0 | 0 | 0 |
| Classifier | None | None | None |
| Class List | None | None | None |
| Hue Value | 0.62 | 0.95 | 0.8 |
| Hue Width | 0.4 | 0.23 | 0.5 |
| Colour Saturation | 0.005 | 0.1 | 0.04 |
| Iwp(High) | 220 | 220 | 255 |
| Iwp(Low) = Ip(High) | 175 | 175 | 200 |
| Ip(Low) = Isp(High) | 100 | 100 | 100 |
| Isp(Low) | 0 | 0 | 0 |
| Inp(High) | -1 | -1 | -1 |

**Table S3.** Cell surface markers used to define key immune cell populations in the tissues examined.

| **Cell type** | **Tissues** | **Gating** |
| --- | --- | --- |
| CD3^bright^ γδ T cells | Lung | CD45^+^ γδTCR^+^ CD3^bright^ |
| Conventional γδ T cells | IEL, LP, Lung | CD45^+^ γδTCR^+^ CD3^+^ |
| CD4^+^ T cells | IEL, LP, Lung | CD45^+^ γδTCR^-^ CD8^-^ CD4^+^ |
| CD8^+^ T cells | IEL, LP, Lung | CD45^+^ γδTCR^-^ CD4^-^ CD8^+^ |
| Eosinophils | IEL, LP, Lung, Spleen | CD45^+^ CD11b^+^ Ly6G^-^ Siglec-F^+^ |
| Neutrophils | IEL, LP, Lung, Spleen | CD45^+^ CD11b^+^ Siglec-F^-^ Ly6G^+^ |

**Fig. S1.** Profile of the indicated cytokines, growth factors and chemokines in the serum of 12-wk-old SHIP-1^+/-^ mice (+/-), SHIP-1^-/-^ mice without ileitis (-/-N), and SHIP-1^-/-^ mice with ileitis (-/-I). Data is presented as median ± IQR. ns = not significant; * *P* < 0.05; ** *P* < 0.01; *** *P* < 0.001; **** *P* < 0.0001 by Kruskal-Wallis test. Non-significant differences are unmarked.

**
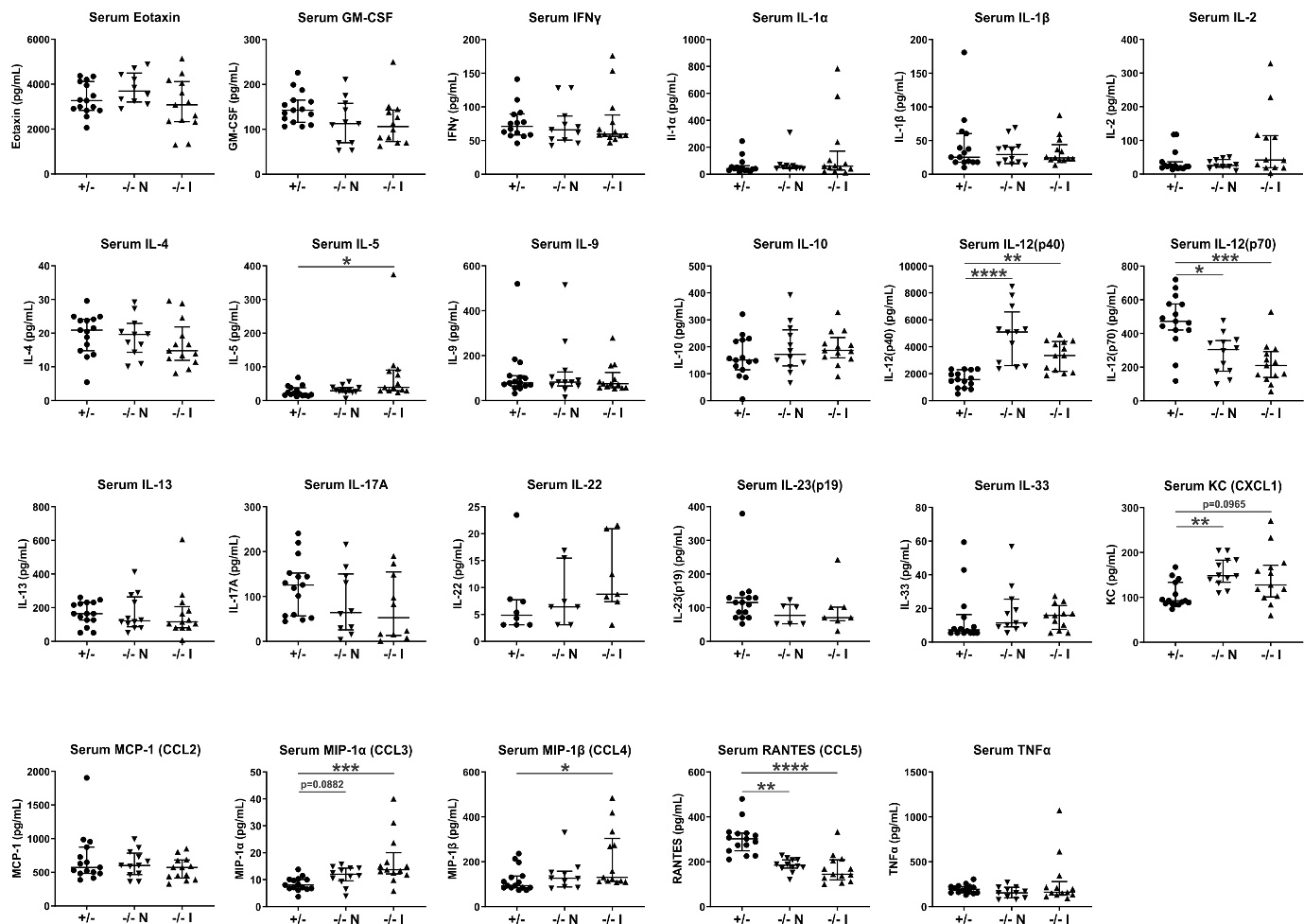
**

**Fig. S2.** Relative abundance of *Bifidobacterium* ASVs in 12-week-old SHIP-1^+/-^ mice, SHIP-1^-/-^ mice without ileitis, and SHIP-1^-/-^ mice with ileitis. Differential abundance testing using a Zero-inflated Gaussian mixture model (metagenomeSeq) showing CSS-normalised relative abundance per group of all *Bifidobacterium* ASVs in dataset. Data is presented as median + SE with statistics representing the *FDR*-corrected *P*-value; n.s. is not significant; * FDR < 0.05; ** FDR < 0.01.


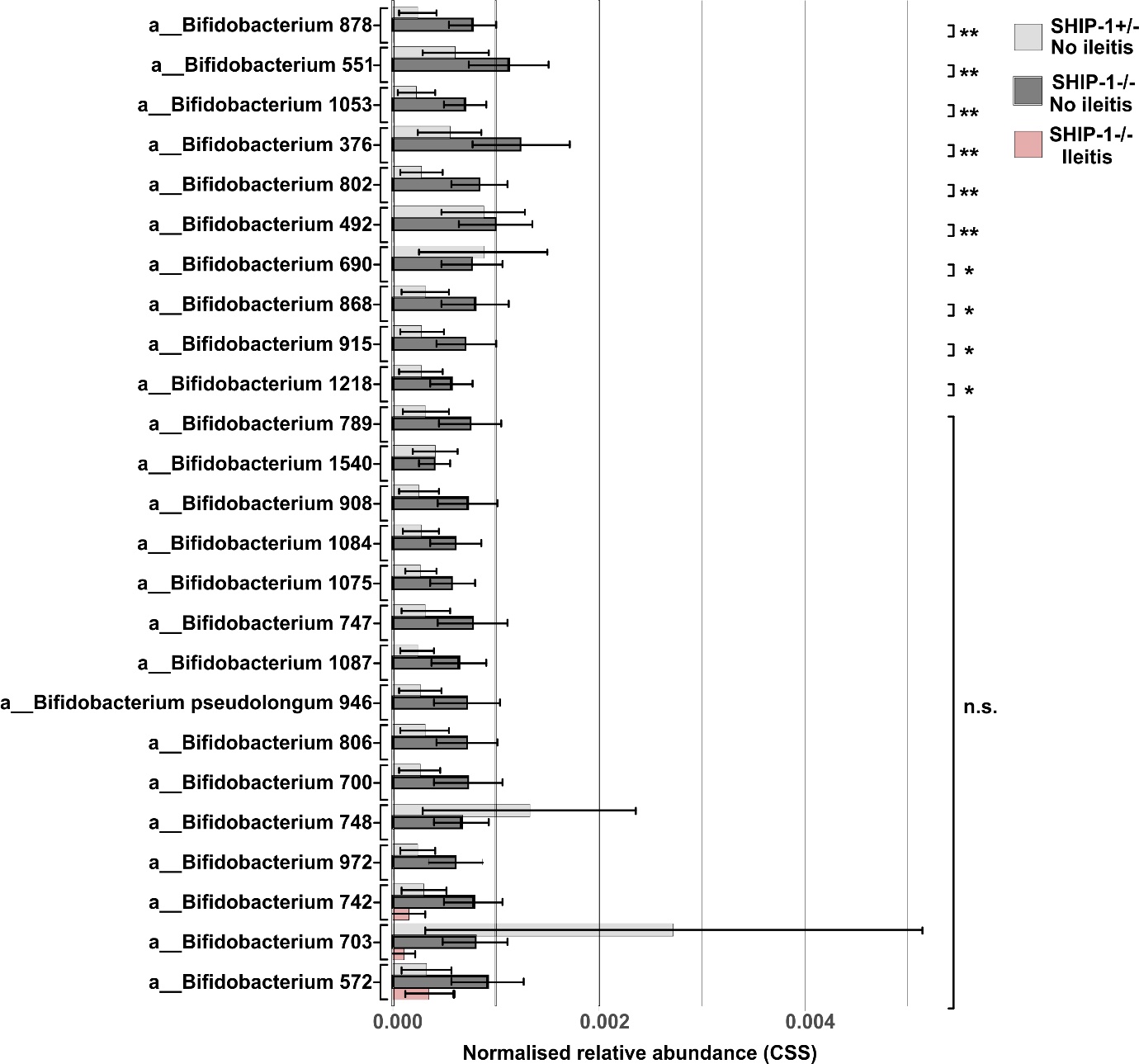


**Fig. S3.** Profile of the indicated cytokines, growth factors and chemokines in the BAL fluid of 12-wk-old SHIP-1^+/-^ mice (+/-), SHIP-1^-/-^ mice without ileitis (-/-N), and SHIP-1^-/-^ mice with ileitis (-/-I). Data is presented as median ± IQR. ns = not significant; * *P* < 0.05; ** *P* < 0.01; *** *P* < 0.001; **** *P* < 0.0001 by Kruskal-Wallis test. Non-significant differences are unmarked.


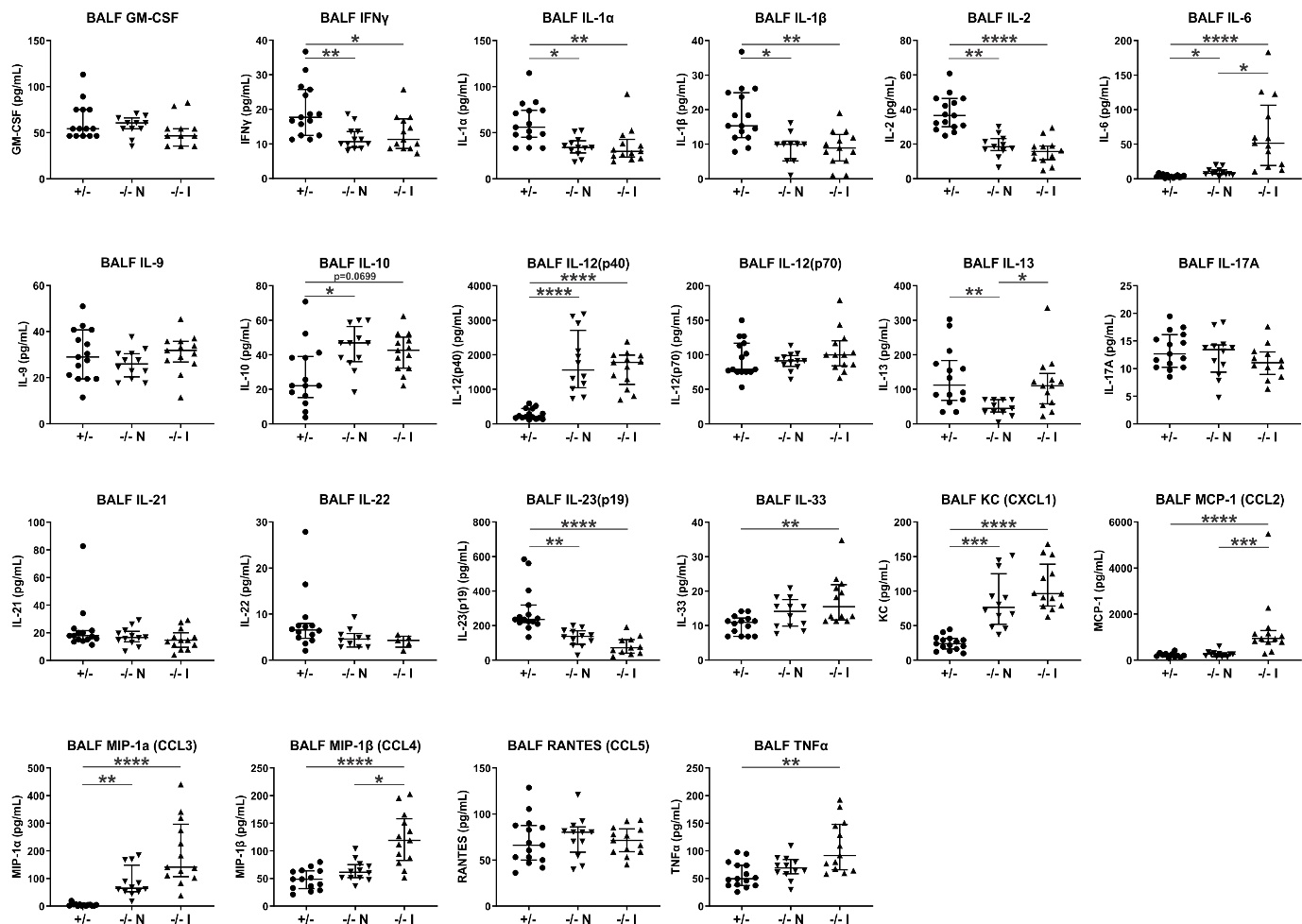

Supplement: Supplementary file 1 — Supplementary Material 1 [file 11_2024_1953_MOESM1_ESM.docx]
